# The SCD1 inhibitor aramchol interacts with regorafenib and metformin to kill tumor cells

**DOI:** 10.18632/oncotarget.28861

**Published:** 2026-03-27

**Authors:** Michael R. Booth, Laurence Booth, Jane L. Roberts, John M. Kirkwood, Paul Dent

**Affiliations:** ^1^Department of Biochemistry and Molecular Biology, Virginia Commonwealth University, Richmond, VA 23298, USA; ^2^Melanoma and Skin Cancer Program, Hillman Cancer Research Pavilion Laboratory, University of Pittsburgh Cancer Institute, Pittsburgh, PA 15213, USA

**Keywords:** macroautophagy, ER stress, aramchol, regorafenib, BID

## Abstract

Mechanisms by which the Stearoyl-CoA desaturase (SCD1) inhibitor aramchol kills tumor cells have recently been described, demonstrating that enhanced signaling through the AMPK played a key role in the processes regulating cell death. Metformin is an anti-hyperglycemic drug which utilizes AMPK signaling to reduce plasma glucose levels. The primary site of metastatic spread of uveal melanoma (UM) is the liver and aramchol concentrates in the liver compared to plasma and other tissues. Aramchol and metformin interacted to modestly enhance cell death in PDX UM cells, though this was less than that caused by the combination of aramchol and the multi-kinase inhibitor regorafenib. Metformin significantly enhanced killing by aramchol plus regorafenib. Metformin significantly enhanced autophagosome formation and autophagic flux caused by aramchol plus regorafenib. Knock down of Beclin1, ATG5 or LAMP2 reduced autophagosome and autolysosome formation, and tumor cell killing. Knock down of BID further enhanced the protective effect of Beclin1 knock down. Knock down of SCD1 enhanced the percentage of dead cells in vehicle control treated cells but did not alter the abilities of drugs to kill tumor cells. Our data demonstrates that UM cells are killed by treatment with aramchol plus regorafenib plus metformin via enhanced autophagic flux and that this combination may have the potential to control UM tumors that have metastasized to the liver.

## INTRODUCTION

The Stearoyl-CoA desaturase (SCD1) inhibitor aramchol was initially developed for patients with Metabolically Associated steatohepatitis (MASH) [1]. It concentrates in the liver over other tissues and within the liver is over 100 μM at steady state [2]. Aramchol both enzymatically inhibits SCD1 and in parallel over time reduces SCD1 protein levels [3, 4]. The loss of SCD1 results in enhanced fatty acid oxidation and increased glutathione levels, hence stabilizing the redox status of cells [5]. Thus, aramchol acts to reduce liver fibrosis and improve the performance status of MASH patients [6].

Our first series of studies with aramchol had defined portions of its anti-tumor mechanisms as a single agent and when combined with the multi-kinase inhibitor regorafenib [7]. Aramchol interacted with the multi-kinase inhibitors sorafenib, regorafenib or lenvatinib, with regorafenib exhibiting the greatest effect. In HCT116 cells homozygous for the macroautophagy-regulatory protein ATG16L1 T300, aramchol and regorafenib interacted to activate ATM and the AMPK and to inactivate mTORC1 and mTORC2. As a single agent, regorafenib inactivated eIF2α, i.e., an endoplasmic reticulum stress response, and it combined with aramchol to elevate expression of the chaperone GRP78. In HCT116 cells homozygote for expressing the ATG16L1 A300 isoform the drug-induced dephosphorylation of mTORC1 S2448 and mTORC2 S2481 and the increased phosphorylation of eIF2α S51 were significantly lower than in cells homozygote for ATG16L1 T300. In cells expressing ATG16L1 T300, but not A300, regorafenib and/or the drug combination inactivated AKT, ERK1/2 and p70 S6K.

Regorafenib and aramchol interacted to cause formation of autophagosomes, which was significantly greater in cells homozygous for ATG16L1 T300 compared to ATG16L1 A300. Aramchol as a single agent did not stimulate autophagic flux but further enhanced both flux and autolysosome formation caused by regorafenib. Knock down of Beclin1 or ATG5 reduced the lethality of regorafenib and aramchol as single agents and when combined whereas knock down of LAMP2 or BID did not reduce killing caused by aramchol as a single agent but did reduce the lethality of regorafenib as a single agent and treatment with regorafenib plus aramchol. *In vivo*, using the HuH7 adult hepatoma cell line in an established-tumor flank model, regorafenib and aramchol interacted to suppress tumor growth without any obvious normal tissue toxicities in the mouse [7].

Uveal melanoma (UM) is a rare cancer of the eye with an incidence of approximately 1 person per 100,000 of population [8]. Recently, the FDA approved the drug Kimmtrak (Tebentafusp) for a specific subset of UM patients expressing the tissue type HLA-A^*^02:01; however for most patients, there is no good therapeutic intervention and clinical trials are recommended [9]. In approximately 90% of UM patients have driving mutations in G alpha proteins that, like mutant RAS proteins in other tumor types, have lost their GTPase activity [10–14] In approximately 50% of UM patients, BRCA1 associated protein-1, BAP1, (ubiquitin carboxy-terminal hydrolase), a deubiquitinating enzyme, is mutated inactive, i.e., BAP1 is a tumor suppressor, and its loss of function subsequently was also associated with BAP1 acting as a suppressor of metastatic spread [15–18]. The site of metastasis for UM is most frequently observed in the liver, and it is the major site of morbidity and mortality for UM patients [19, 20]. Anti-cancer drugs which tend to concentrate in the liver, such as aramchol, may thus have therapeutic utility in treating UM patients.

Metformin is primarily used to treat type 2 diabetics [21–23]. One mechanism of metformin action is to activate the AMPK. In our prior work, the anti-tumor actions of aramchol required activation of the AMPK, which was upstream of autophagosome formation [7]. We hypothesized that tumor cells exposed to both aramchol and metformin would exhibit hyper-activation of AMPK signaling, resulting in greater levels of macroautophagy and tumor cell killing. The present studies were designed to test the hypothesis that metformin will enhance the lethality of aramchol plus regorafenib.

## RESULTS

**Figure 1:**
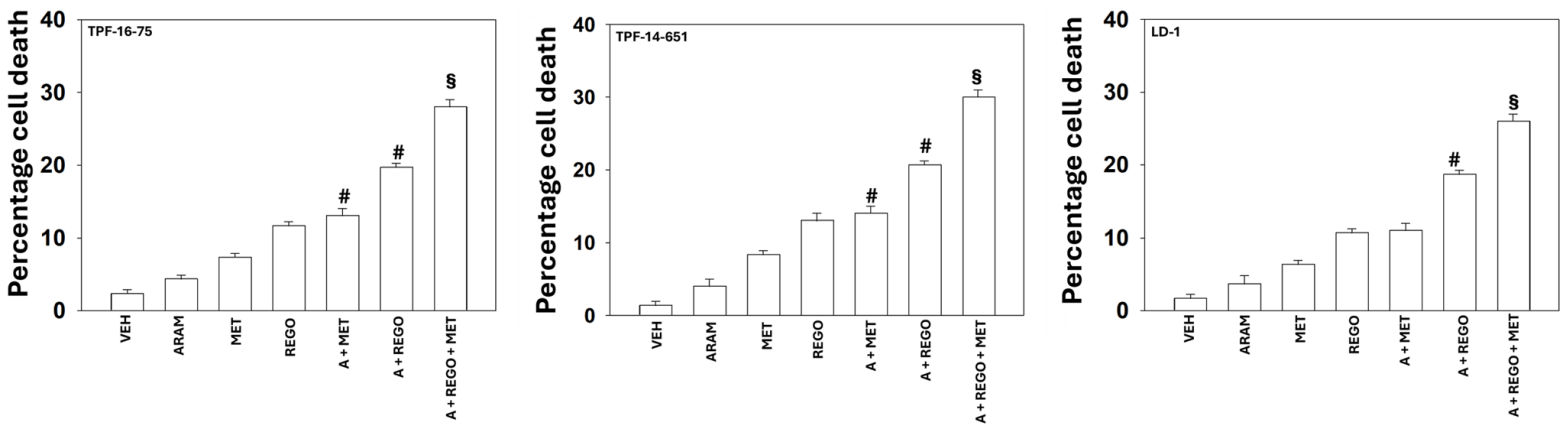
Aramchol, metformin and regorafenib interact to kill uveal melanoma and cholangiocarcinoma cells. PDX uveal melanoma cells and LD-1 cholangiocarcinoma cells were treated with vehicle control, aramchol, regorafenib, metformin or the drugs combined as indicated for 24 h. Floating and attached cells from three independent studies were collected and the percentage viability determined using trypan blue exclusion assays (±SD). ^§^*p* < 0.05 greater than all other drug treated cells; ^#^*p* < 0.05 greater than cells treated with one drug.

Our initial studies determined the impact of aramchol, regorafenib and metformin on signaling within tumor cells and whether metformin enhanced the lethality of aramchol plus regorafenib in patient-derived UM cells, and in a cholangiocarcinoma cell line LD-1. Metformin interacted with aramchol and with aramchol plus regorafenib to enhance tumor cell killing in UM and cholangiocarcinoma cells (Figure 1). Similar data were obtained in GI-derived tumor cell lines (Supplementary Figure 1).

The impact of drug exposure on cell signaling, 4 h after treatment, was determined in several cell lines. From a collective appraisal of our findings, aramchol, regorafenib and metformin interacted to cause greater activation of ATM, ULK1, and PERK, and greater expression of BAK (Tables 1–3; Supplementary Tables 1–3). The drugs interacted to inactivate mTORC1 and mTORC2, p70 S6K, ERK1/2, ERBB2 and c-MET, and to reduce the expression of MCL1.

**Table 1:** Aramchol, regorafenib and metformin interact to alter the phosphorylation and expression of proteins

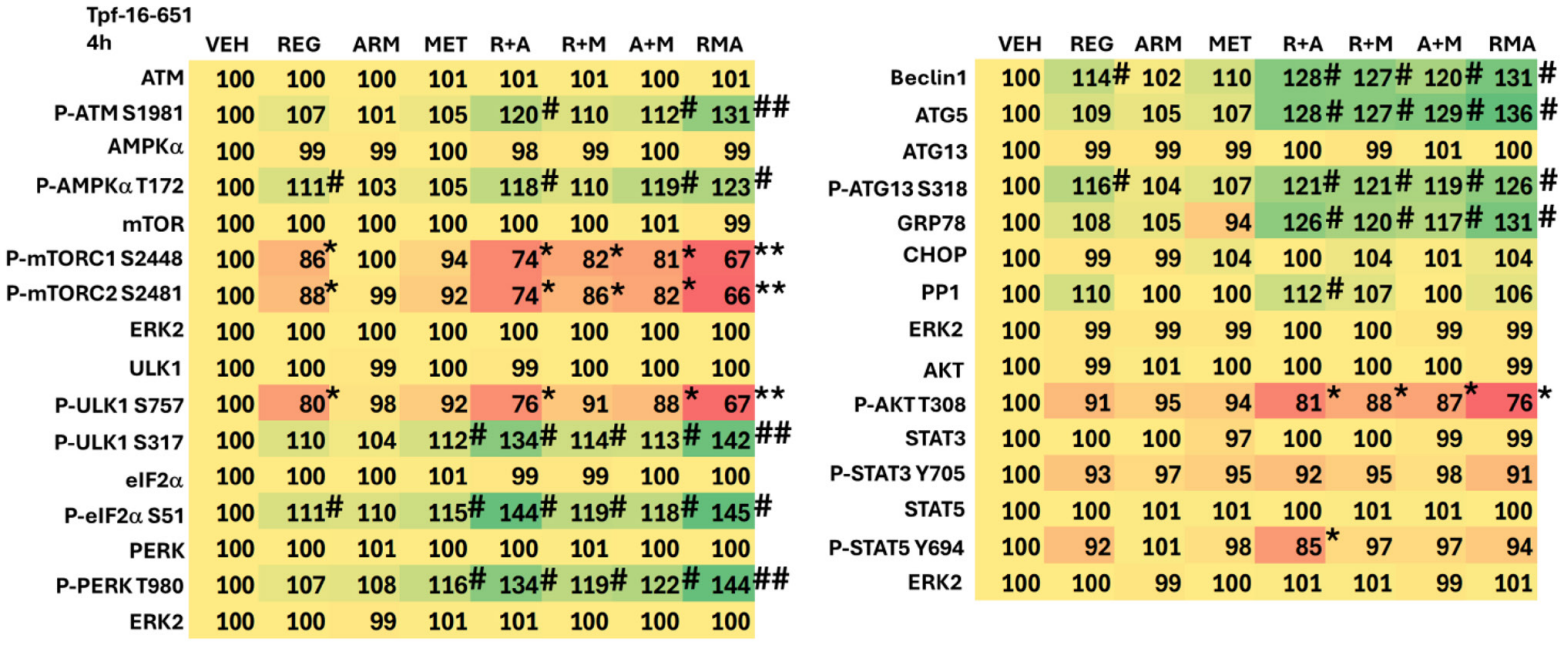

TPF-16-651 PDX UM cells in 96-well plates were treated with vehicle control, aramchol, regorafenib, metformin or the drugs combined for 4 h. Cells were fixed in place, permeabilized and subjected to in-cell immunostaining for the indicated proteins/phospho-proteins. Cells were imaged using an Odyssey infrared imager. ERK2 staining was used as an invariant loading control. The percentage alteration in expression/phosphorylation caused by the drugs was determined from three independent replicates (±SD). ^#^*p* < 0.05 greater than cells treated with one drug; ^##^*p* < 0.05 greater than cells treated with two drugs; ^*^p < 0.05 less than vehicle control; ^**^p < 0.05 less than cells treated with two drugs.

**Table 2:** Aramchol, metformin and regorafenib interact to alter the phosphorylation and expression of proteins

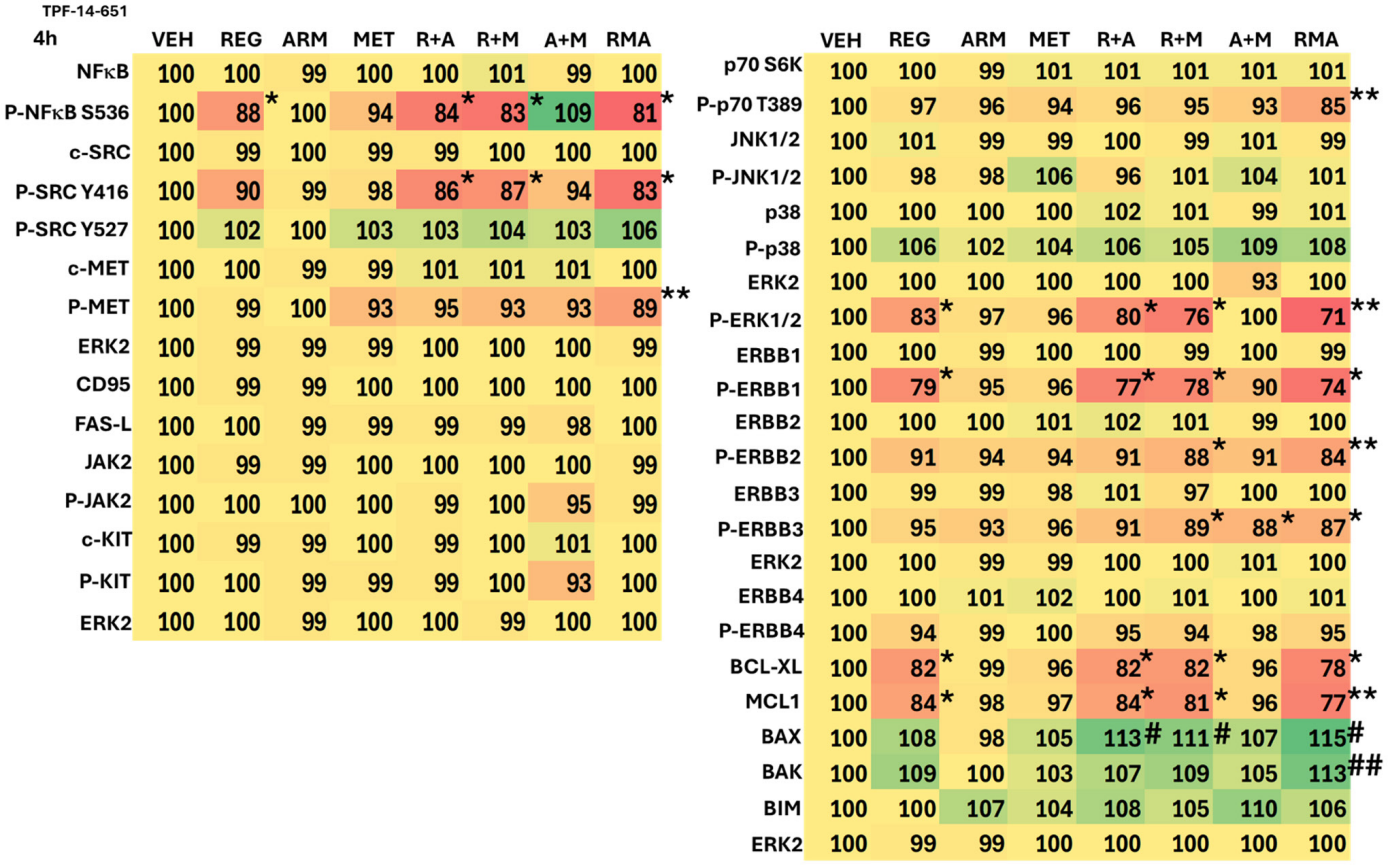

TPF-16-651 PDX UM cells in 96-well plates were treated with vehicle control, aramchol, regorafenib, metformin or the drugs combined for 4 h. Cells were fixed in place, permeabilized and subjected to in-cell immunostaining for the indicated proteins/phospho-proteins. Cells were imaged using an Odyssey infrared imager. ERK2 staining was used as an invariant loading control. The percentage alteration in expression/phosphorylation caused by the drugs was determined from three independent replicates. (±SD). ^#^*p* < 0.05 greater than cells treated with one drug; ^##^*p* < 0.05 greater than cells treated with two drugs; ^*^p < 0.05 less than vehicle control; ^**^p < 0.05 less than cells treated with two drugs.

**Table 3:** Aramchol, metformin and regorafenib interact to alter the phosphorylation and expression of proteins

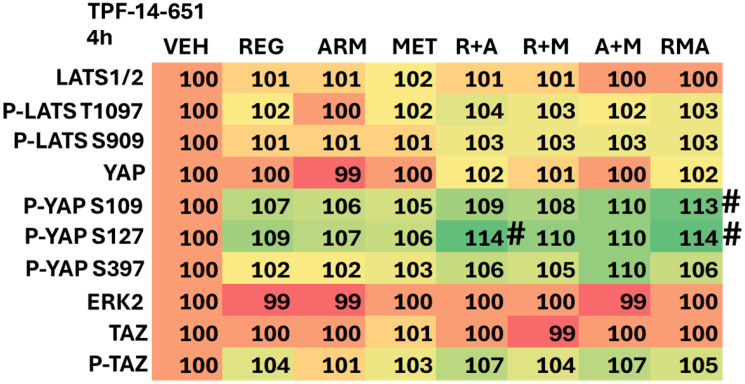

TPF-16-651 PDX UM cells in 96-well plates were treated with vehicle control, aramchol, regorafenib, metformin or the drugs combined for 4 h. Cells were fixed in place, permeabilized and subjected to in-cell immunostaining for the indicated proteins/phospho-proteins. Cells were imaged using an Odyssey infrared imager. ERK2 staining was used as an invariant loading control. The percentage alteration in expression/phosphorylation caused by the drugs was determined from three independent replicates (±SD). ^#^*p* < 0.05 greater than cells treated with one drug; ^##^*p* < 0.05 greater than cells treated with two drugs; ^*^p < 0.05 less than vehicle control; ^**^p < 0.05 less than cells treated with two drugs.

In addition to interactions at the three-drug combination level, two-drug interactions were also observed, most notably those combining aramchol plus regorafenib. Here, we observed the activation of ATM, AMPK, ULK1, and PERK, and increased expression of Beclin1, ATG5, GRP78, PP1 and BAX. Inactivation was found for mTORC1, mTORC2, eIF2α, AKT, STAT5, and YAP (Tables 1–3; Supplementary Tables 1–3). As a single agent, regorafenib more potently altered signaling and expression than either aramchol or metformin. All these alterations would, a priori, predict for tumor cells to exhibit higher levels of autophagosomes and enhanced mitochondrial dysfunction.

**Figure 2:**
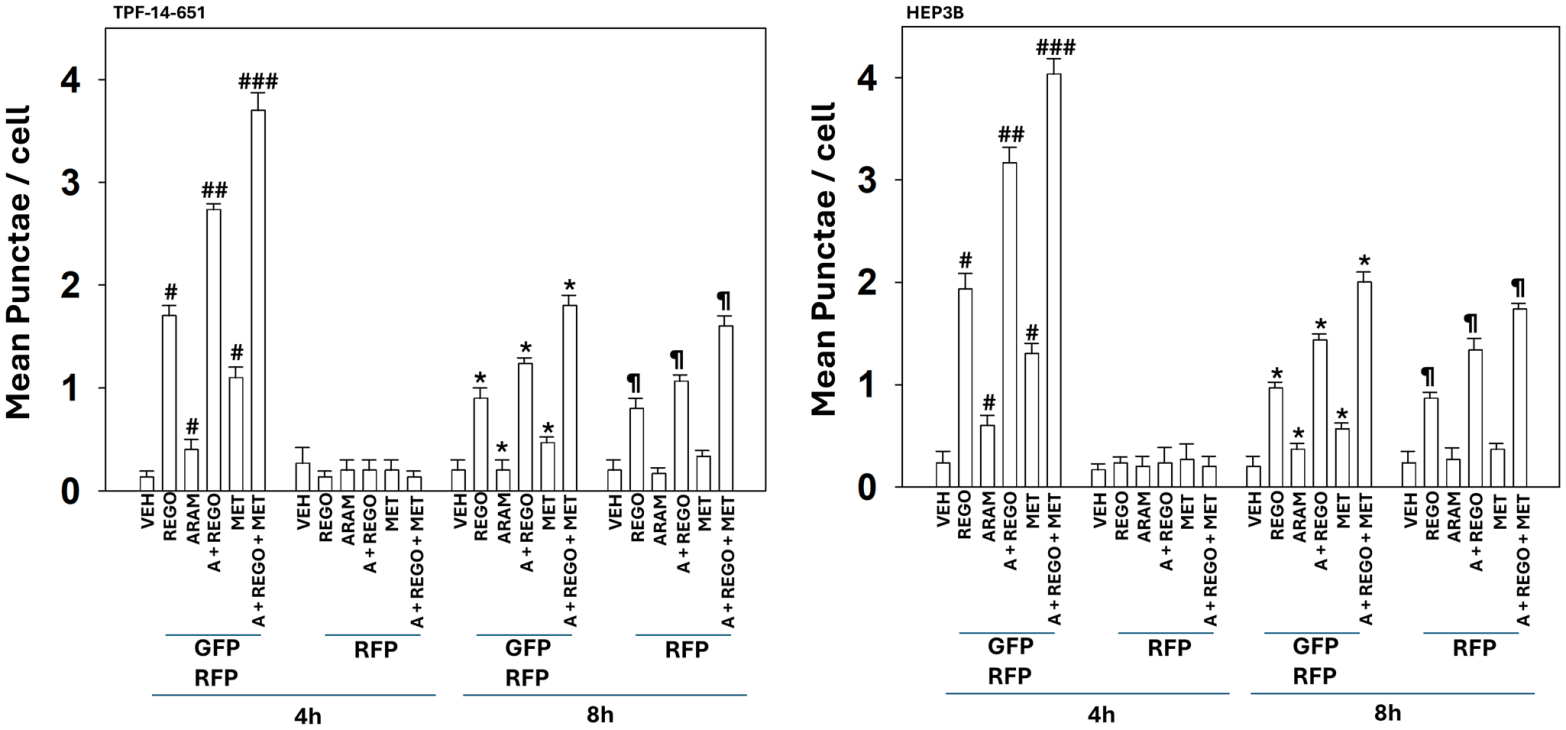
The aramchol plus regorafenib combination causes the formation of autophagosomes and autolysosomes which is enhanced by metformin. TPF-14-651 UM and HEP3B HBV-infected hepatoma cells were transfected with a plasmid to express LC3-GFP-RFP. After 24 h, cells were treated with vehicle control, aramchol, regorafenib, metformin or the drugs combined for 4 h and 8 h. At each time point, the mean numbers of intense [GFP + RFP] and [RFP alone] vesicles from 100 randomly selected cells were determined (*n* = 3 ± SD). ^#^*p* < 0.05 greater than vehicle control; ^##^*p* < 0.05 greater than drugs treated with a single agent; ^###^*p* < 0.05 greater than drugs treated with two agents; ^*^p < 0.05 less than corresponding values at the 4 h time point; ^¶^*p* < 0.05 less than corresponding values at the 4 h time point.

To measure macroautophagy and autophagic flux, we made use of a plasmid that encodes for expression of a fusion protein, LC3-GFP-RFP. In the early autophagosome, both GFP and RFP fluoresce, and yellow vesicles are observed. After the fusion of the autophagosome with a lysosome, followed by its acidification, an autolysosome can be detected examining the numbers of red vesicles, as GFP fluorescence is quenched under acidic conditions. Our prior studies had mechanistically linked tumor cell killing caused by aramchol plus regorafenib to enhanced formation of autophagosomes and autolysosomes [7]. Both regorafenib as a single agent and aramchol plus regorafenib significantly enhanced the formation of autophagosomes followed by the formation of autolysosomes, i.e., autophagic flux (Figure 2). The effects of metformin on macroautophagy as a single agent were significantly lower than those of regorafenib. However, metformin significantly enhanced aramchol plus regorafenib-stimulated autophagosome and autolysosome formation.

**Figure 3:**
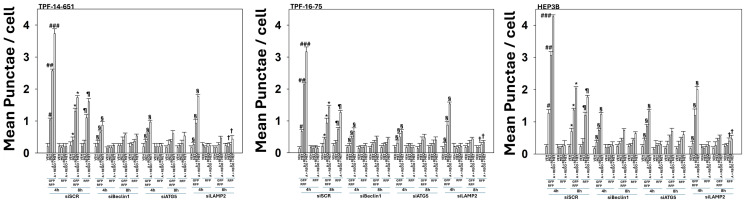
Aramchol, metformin and regorafenib enhance the formation of autophagosomes and autolysosomes which are reduced by knock down of Beclin1, ATG5 or LAMP2. UM cells and HEP3B cells were transfected with a scrambled siRNA (siSCR) or with siRNA molecules to knock down the expression of Beclin1, ATG5 or LAMP2. In parallel, cells were transfected with a plasmid to express LC3-GFP-RFP. After 24 h, cells were treated with vehicle control, aramchol, regorafenib, metformin or the drugs combined for 4 h and 8 h. At each time point, the mean numbers of intense [GFP + RFP] and [RFP alone] vesicles from 100 randomly selected cells were determined (*n* = 3 ± SD). ^#^*p* < 0.05 greater than vehicle control; ^##^*p* < 0.05 greater than drugs treated with a single agent; ^###^*p* < 0.05 greater than drugs treated with two agents; ^*^p < 0.05 less than corresponding values at the 4 h time point; ^¶^*p* < 0.05 less than corresponding values at the 4 h time point; ^§^*p* < 0.05 less than corresponding values in siSCR cells; ^†^*p* < 0.05 less than corresponding values in siBeclin1 and siATG5 cells.

**Figure 4:**
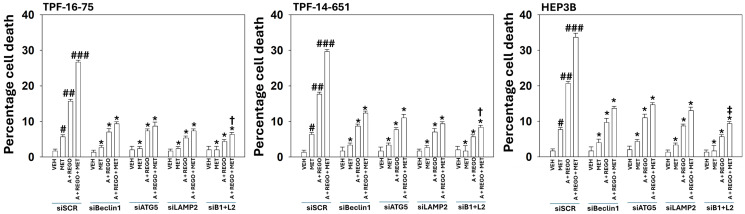
Knock down of Beclin1 and LAMP2 significantly reduces the lethality of [aramchol and regorafenib and metformin]. UM cells and HEP3B cells were transfected with a scrambled siRNA (siSCR) or with siRNA molecules to knock down the expression of Beclin1, ATG5 or LAMP2. After 24 h, cells were treated with vehicle control, aramchol, regorafenib, metformin or the drugs combined for 24 h. Floating and attached cells from three independent studies were collected and the percentage viability determined using trypan blue exclusion assays (±SD). ^#^*p* < 0.05 greater than vehicle control; ^##^*p* < 0.05 greater than drugs treated with a single agent; ^###^*p* < 0.05 greater than drugs treated with two agents; ^*^p < 0.05 less than corresponding values in siSCR transfected cells; ^†^*p* < 0.05 less than corresponding values in siBeclin1 cells; ^‡^*p* < 0.05 less than corresponding values in siBeclin1 and siATG5 cells.

Knock down of Beclin1 or ATG5 significantly reduced the formation of autophagosomes and autolysosomes (Figure 3). Knock down of LAMP2, an essential protein for autolysosome formation reduced autophagosome formation and abolished autolysosome formation. We next determined the impact of the siRNA manipulations used in Figure 3 on the amount of tumor cell killing we had previously observed in Figure 1. Knock down of Beclin1, ATG5 or LAMP2 significantly reduced tumor cell killing by all agents, regardless of their combinations (Figure 4). Combined knock down of Beclin1 and LAMP2 caused a significant further decline in tumor cell death.

**Figure 5:**
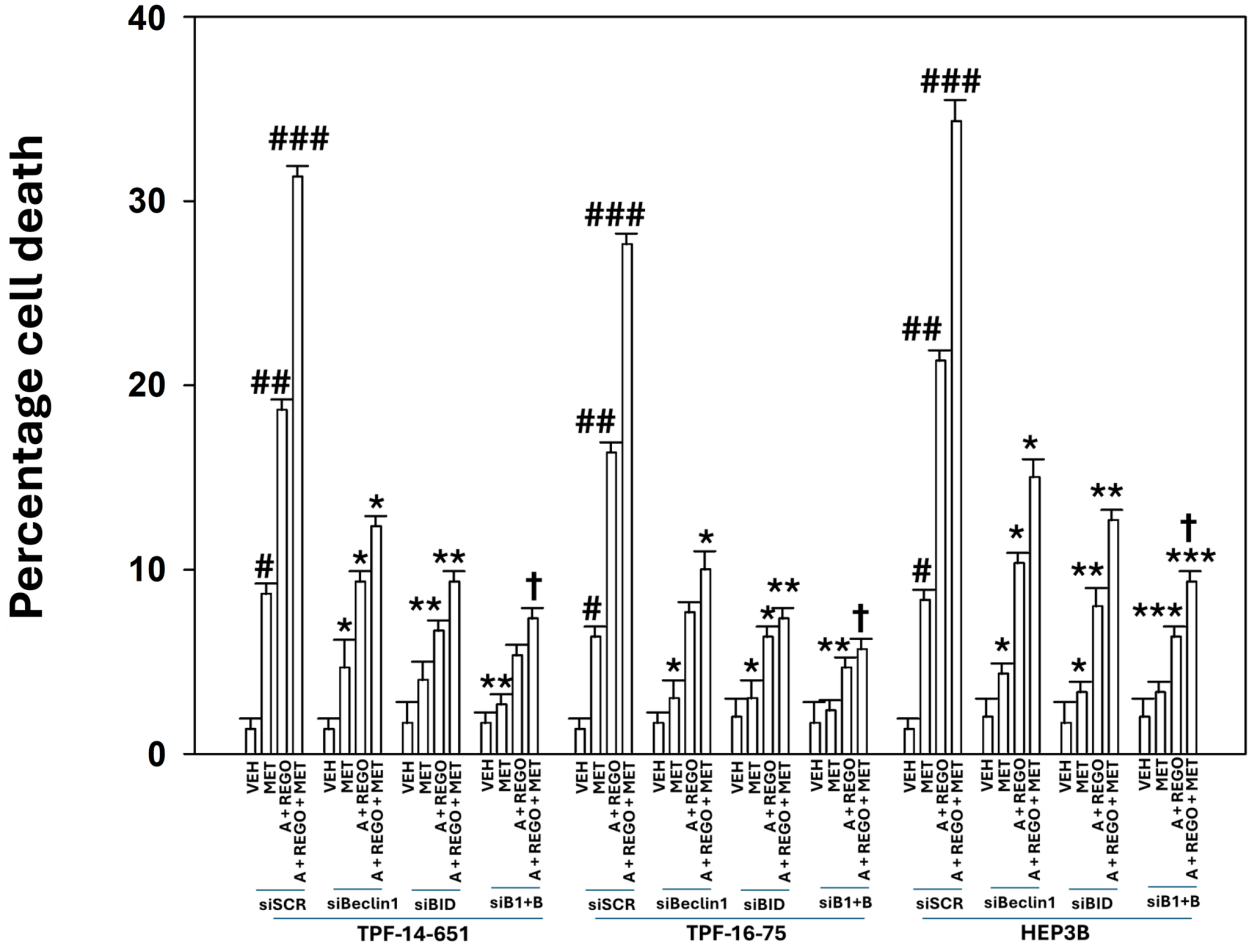
Metformin increases tumor cell killing caused by [aramchol and regorafenib], which requires macroautophagy and signaling through BID. UM cells and HEP3B cells were transfected with a scrambled siRNA control (siSCR) or with siRNA molecules to knock down Beclin1 and BID. After 24 h, cells were treated with vehicle control, aramchol, regorafenib, metformin or the drugs combined for 24 h. Floating and attached cells from three independent studies were collected and the percentage viability determined using trypan blue exclusion assays (±SD). ^#^*p* < 0.05 greater than vehicle control; ^##^*p* < 0.05 greater than drugs treated with a single agent; ^###^*p* < 0.05 greater than drugs treated with two agents; ^*^p < 0.05 less than corresponding values in siSCR transfected cells; ^**^p < 0.05 less than corresponding value in siBeclin1 cells; ^†^*p* < 0.05 less than corresponding values in siBID cells.

Our prior work demonstrated that aramchol plus regorafenib utilized death receptor signaling as a portion of the mechanism by which the drug combination killed tumor cells [7]. Death receptors signal through caspase 8 and BID to cause mitochondrial dysfunction, and tumor cell death. Knock down of BID reduced tumor cell killing caused by metformin, aramchol plus regorafenib and the three drug combination (Figure 5). Hence, as predicted from our signal transduction analyses, tumor cell killing by aramchol plus regorafenib plus metformin is a multi-factorial process requiring both enhanced macroautophagy and flux, and death receptor signaling via BID.

**Figure 6:**
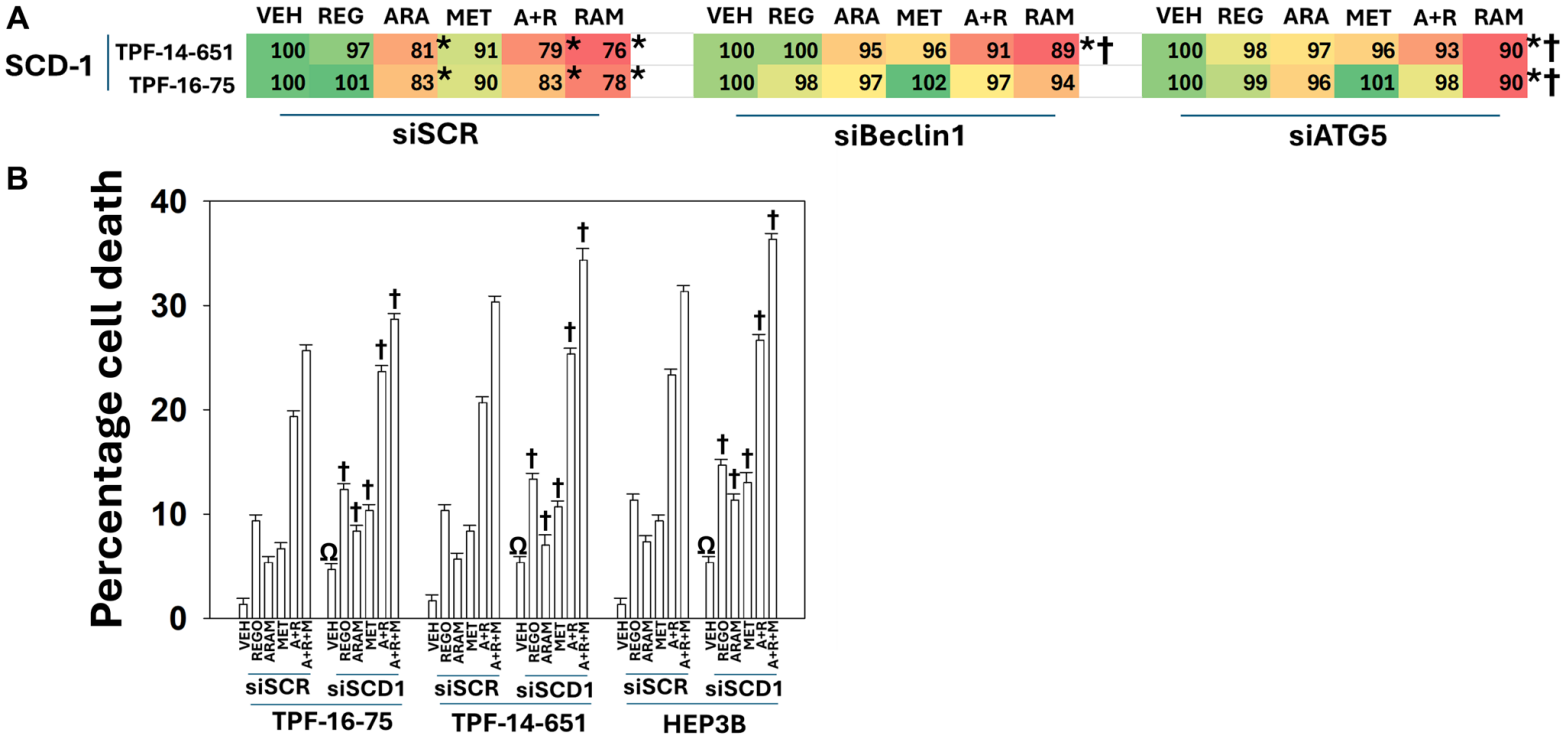
Down-regulation of SCD1 by aramchol requires macroautophagy. (**A**) UM cells were transfected with a scrambled siRNA or with siRNA molecules to knock down the expression of Beclin1 or ATG5. After 24 h, cells were treated with vehicle control, aramchol, regorafenib, metformin or the drugs combined for 4 h. Cells were fixed in place, permeabilized and subjected to in-cell immunostaining for SCD1. Cells were imaged using an Odyssey infrared imager. ERK2 staining was used as an invariant loading control. The percentage alteration in expression caused by the drugs was determined from three independent replicates (±SD). ^*^p < 0.05 less than cells treated with vehicle control. ^†^*p* < 0.05 less degradation of SCD1 compared to the corresponding values in siSCR cells. (**B**) UM and HEP3B cells were transfected with a scrambled siRNA or with an siRNA molecule to knock down the expression of SCD1. After 24 h, cells were treated with vehicle control, aramchol, regorafenib, metformin or the drugs combined for 24 h. Floating and attached cells from three independent studies were collected and the percentage viability determined using trypan blue exclusion assays (±SD). ^Ω^*p* < 0.05 greater than vehicle control value in siSCR transfected cells; ^†^*p* < 0.05 greater than treatment with the same agent in siSCR cells.

We next examined the expression of the stated aramchol target, SCD1, in UM cells. Treatment of UM cells with aramchol significantly reduced SCD1 expression (Figure 6A). Combined treatment with aramchol, regorafenib and metformin had no significant additional effect on reducing SCD1 levels, below those caused by aramchol though a non-significant trend was observed. Knock down of Beclin1 or ATG5 prevented aramchol as a single agent and when combined with regorafenib or metformin from reducing SCD1 expression. Hence, although regorafenib causes significantly greater levels of autophagosome formation than does aramchol, SCD1 could only be degraded by macroautophagy when it had first been “*destabilized*” by the actions of aramchol.

Using a validated siRNA molecule to knock down the expression of SCD1, we determined whether all of the anti-tumor actions of aramchol could be ascribed only to the selective inhibition of SCD1 or whether aramchol has multiple unknown targets that collectively are components of aramchol anti-tumor biology (Figure 6B). Knock down of SCD1, in vehicle control treated cells, significantly increased basal levels of cell death from ~1.5% to ~5% (absolute level). In cells with knock down of SCD1 expression, the lethality of regorafenib, aramchol, metformin and when combined was also ~4% higher than the same value observed in scrambled siRNA transfected cells. Of note, treatment of siSCD1 transfected cells with regorafenib did not phenocopy its cell killing amount to that of siSCR transfected cells treated with regorafenib plus aramchol. These findings collectively argue that for its anti-cancer effects, there are additional targets of aramchol beyond SCD1.

## DISCUSSION

The present investigations were developed as an extension to our earlier work in GI tumor cells combining the SCD1 inhibitor aramchol with the multi-kinase inhibitor regorafenib. We determined that the AMPK played a key role in promoting toxic macroautophagy, and hence we determined whether metformin, a drug used for diabetics that activates the AMPK, could enhance the anti-tumor actions of aramchol plus regorafenib. The primary hypothesis of the work was subsequently validated, showing that metformin enhanced the anti-cancer effects of aramchol plus regorafenib.

We have demonstrated in tumor cells with a wide range of anti-cancer drugs that ATM becomes activated, in part due to the actions of reactive oxygen species, and not DNA damage, and as a result of this, cells exhibit enhanced T172 phosphorylation of AMPKα [7, 24–27]. We observed that aramchol plus regorafenib caused ATM-dependent increased phosphorylation of AMPKα T172, which was required for altering the regulatory phosphorylation of mTORC1 S2448, mTORC2 S2481, ULK1 S317/S757 and the autophagy-gatekeeper ATG13 S318 [7].

In our cell signaling analyses, regardless of tumor cell type regorafenib caused inactivation of mTORC1 and mTORC2, and activation of ULK1, and when combined with either aramchol or metformin, a trend of further mTOR inactivation and ULK1 activation was observed. Only with the three-drug combination of regorafenib, aramchol and metformin did we observe a further significant inaction of mTORC1 and mTORC2 and activation of ULK1. These events were associated with greater amounts of autophagosome formation, which played an essential role in the mechanism of tumor cell killing. In further support for a key mechanism of killing relying on the regulation of signaling through the PI3K pathway was that the three-drug combination in UM and hepatoma cells was required to inactivate ERBB3, AKT and p70 S6K.

As noted in the prior paragraph, we observed greater activation of ULK1 with the three-drug combination, however, this was not reflected in the phosphorylation of the direct target of ULK1, the autophagy-gatekeeper ATG13 S318. On the other hand, as predicted by greater ULK1 activation and greater mTORC1 inactivation, we did observe elevated levels of autophagosome formation. This data suggests that the three-drug combination is regulating the function and/or expression of other proteins, i.e., there are also evidently unknown effector pathways being regulated which also contribute to enhanced macroautophagy and tumor cell killing.

One possible mechanism by which this ATG13 S318 -independent mechanism could have occurred was via ER stress signaling and its control of Beclin1 and ATG5 expression. In prior studies in multiple tumor cell types, we have shown that PERK-eIF2α signaling acted to increase expression of Beclin1 and ATG5 [24–27]. In our present studies, we observed the three-drug combination causing greater activation of PERK, though this effect was not significant or consistent at the level of eIF2α or for increased expression of GRP78, Beclin1 or ATG5.

In our prior work with aramchol and regorafenib, we discovered that combined knock down of Beclin1 and LAMP2 or knock down of the caspase 8 substrate BID significantly reduced the amount of tumor cell death caused by aramchol plus regorafenib [7]. In the present studies, knock down of Beclin1 reduced the formation of autophagosomes and autolysosomes, and knock down of LAMP2 almost abolished autolysosome formation. Combined knock down of Beclin1 and LAMP2 abolished tumor cell killing by metformin, and significantly reduced killing caused by aramchol plus regorafenib and the three drug combination below that caused by Beclin1 knock down alone. Knock down of BID significantly reduced the lethality of metformin as a single agent, aramchol plus regorafenib and the three drugs combined. Knock down of Beclin1 and BID abolished tumor cell killing caused by metformin. Collectively, this data demonstrates that metformin enhances the killing potential of aramchol plus regorafenib through the already-established mechanisms of macroautophagy and BID cleavage. Future studies beyond the present manuscript will be required to define whether BID cleavage is due to the actions of death receptors/caspase 8 or to cathepsin/calpain enzymes which are also capable of causing BID cleavage.

Aramchol as a single agent reduced the expression of SCD1 that was not significantly altered by combined treatment with regorafenib and metformin. Knock down of Beclin1 or ATG5 abolished the effect of aramchol plus regorafenib on reducing SCD1 levels and significantly reduced actions of aramchol plus regorafenib plus metformin. During prior studies of therapeutic agents, e.g., AR12, sorafenib, neratinib, we determined that a considerable proportion of the anti-tumor effects of each drug were not due to inhibition of the published drug-targets but were instead due to previously unknown proteins, e.g., chaperone proteins for AR12 and sorafenib, MST4, mutant KRAS and NRAS proteins for neratinib [28–31]. Hence, based of prior work and as aramchol is proposed to act through inhibition of SCD1, we determined whether molecular knock down of SCD1 phenocopied to all of the anti-cancer actions of aramchol [1, 7]. Knock down of SCD1, by itself, did modestly reduce the basal levels of tumor cell viability, with absolute death levels go from ~1.5% to ~5%. However, although we see a modest elevation in cell death under basal conditions caused by SCD1 knock down, we did not observe any large alterations in the amounts of killing caused by the drugs combined with SCD1 with knock down. Instead, we observe all death values, under any treatment condition, being elevated upwards by about 4% (absolute value). For example, if aramchol is only “working” by blocking SCD1, and only SCD1, we would expect to observe aramchol plus regorafenib death levels in scrambled control transfected cells being the same as death in knock down of SCD1 and regorafenib; they are not. Collectively, this data links SCD1 to the basal regulation of tumor cell viability, but *not* to the mechanism of aramchol-induced tumor cell killing.

As aramchol was developed to inhibit a bioactive lipid metabolizing enzyme we would predict that the most probable other targets for the agent will also be involved in the regulation of bioactive lipid synthesis and/or degradation. Lipidomic studies in our initial aramchol manuscript showed that aramchol as a single agent increased the levels of ceramide-1-phosphate and of triglycerides [7]. Heat map comparative analyses also demonstrated that the amount of ceramide-1-phosphate and of triglycerides, as well as reduced levels of some phosphatidyl choline species, whilst other species were significantly increased. Additional lipidomic studies with aramchol are to be performed in a future study.

The spread of UM from the eye to distant sites such as the liver results in the progression of morbidity and mortality [32]. Therapeutic combinations of drugs that can reduce or prevent the growth of metastatic UM in the liver environment are urgently needed. Future studies are to be performed in an orthotopic model of UM metastasis to the liver.

## MATERIALS AND METHODS

### Materials

The deidentified PDX uveal melanoma cells TPF-14-651 and TPF-16-75 were kindly provided by Dr. John Kirkwood (Melanoma and Skin Cancer Program, Hillman Cancer Research Pavilion Laboratory, University of Pittsburgh Cancer Institute, Pittsburgh, PA 15213, USA). The hepatoma cell lines HEP3B and HuH7 cells were purchased from Biohippo Inc. (Gaithersburg, MD, USA). HCT116 cells were kindly provided by Dr. David Boone (South Bend, IN, USA). Regorafenib and metformin were purchased from Selleckchem (Houston, TX, USA). Aramchol was provided by Galmed Pharmaceuticals Ltd. (Tel Aviv, Israel). Trypsin-EDTA, DMEM, RPMI, penicillin-streptomycin were purchased from GIBCOBRL (GIBCOBRL Life Technologies, Grand Island, NY, USA). The LC3-GFP-RFP plasmid was obtained from Addgene (Watertown, MA; #117413). Antibodies were from Cell Signaling Technology (Danvers, MA, USA); Abgent (San Diego, CA, USA); Novus Biologicals (Centennial, CO, USA); Abcam (Cambridge, UK); Santa Cruz Biotechnology (Dallas, TX, USA). No human studies were performed as a component of this manuscript [18–21].

### Methods

All bench-side Methods used in this manuscript have been previously performed and described in the peer-reviewed references [18–21]. One problematic issue in cancer developmental therapeutics studies with novel drug combinations is defining the correct concentrations of the agents to be used for *in vitro* assays. Studies by our group over the past ~20 years have used the safely achievable maximal plasma concentration (C max) and protein binding of an agent as guidelines for its concentration range to be used for *in vitro* assays [reference 7, and references therein]. For example, the C max of the multi-kinase inhibitor sorafenib is approximately 13 μM, however because of protein binding, the probable “free” sorafenib concentration will be at or below 2 μM. Similarly, with the multi-kinase inhibitor regorafenib, the probable free concentration of this agent will be less than 1 μM; in our studies, we have therefore used generally used sorafenib (2.0 μM) and regorafenib (0.5 μM). The resting concentration of aramchol in the liver for dosing over 30 days is greater than 100 μM, and for a single dose, the C max is 20 μM. We chose the single dose C max as our working final concentration of aramchol. At steady-state, dosing 850 mg three times daily, the C max of metformin hydrochloride is 12 μM, with our use of metformin at a final concentration of 10 μM. Briefly, cells where indicated were transfected with a scrambled siRNA control (siSCR) or with validated siRNA molecules to knock down the expression of the indicated proteins. After 24 h, cells were treated with vehicle control, [regorafenib (0.5 μM), aramchol (20 μM)], metformin (10 μM) or the drugs combined as indicated for 4 h. Cells were fixed in place, permeabilized and subjected to in-cell immunostaining for the indicated proteins/phospho-proteins. Cells were imaged using an Odyssey infrared imager. The percentage alteration in expression/phosphorylation caused by the drugs was determined from three independent replicates (±SD). Values with a *p* < 0.05 were considered significant.

### Detection of cell death by trypan blue assay

Cells where indicated were transfected with a scrambled siRNA control (siSCR) or with validated siRNA molecules to knock down the expression of the indicated proteins. After 24 h, cells were treated with vehicle control, regorafenib (0.5 μM), aramchol (20 μM)], metformin (10 μM) or the drugs combined for 24 h. At the indicated time points cells were harvested by trypsinization and centrifugation. Cell pellets were resuspended in PBS and mixed with trypan blue agent. Viability was determined microscopically using a hemocytometer. Five hundred cells from randomly chosen fields were counted and the number of dead cells was counted and expressed as a percentage of the total number of cells counted (*n* = 3 ± SD).

### Transfection of cells with siRNA/plasmids

Cells were plated and 24 h after plating, transfected. A plasmid to express LC3-GFP-RFP was used throughout the study (Addgene, Waltham, MA, USA). For siRNA transfection, 10 nM of the annealed siRNA or the negative control (a “scrambled” sequence with no significant homology to any known gene sequences from mouse, rat, or human cell lines) were used. Control studies to define the percentage of protein knock down caused by siRNA molecules are presented in Supplementary Table 4.

### Assessments of autophagosome and autolysosome levels

Cells were transfected with a plasmid to express LC3-GFP-RFP. Twenty-four hours after transfection, After 24 h, cells were treated with vehicle control, regorafenib (0.5 μM), aramchol (20 μM)], metformin (10 μM) or the drugs combined as indicated for 4 h and for 8 h. Cells were imaged at 60X magnification and the mean number of [GFP+RFP+] and [RFP+] punctae per cell determined in living cells from >100 randomly selected cells per condition (*n* = 3 ± SD).

### Data analysis

Data were formatted in SigmaPlot 15.0 to calculate the mean staining intensities ± standard deviation for each protein and phospho-protein. Statistical comparison of the effects between various treatments was made within the SigmaPlot program using one-way ANOVA for normalcy followed by a two tailed Student’s *t*-test with multiple comparisons. Differences with a *p*-value of < 0.05 were considered statistically significant. Experiments are the means of multiple individual data points per experiment from 3 independent experiments (±SD).

## SUPPLEMENTARY MATERIALS



## Abbreviations

ERK: extracellular regulated kinase
PI3K: phosphatidyl inositol 3 kinase
ER: endoplasmic reticulum
AMPK: AMP-dependent protein kinase
mTOR: mammalian target of rapamycin
JAK: Janus Kinase
STAT: Signal Transducers and Activators of Transcription
MAPK: mitogen activated protein kinase
PTEN: phosphatase and tensin homologue on chromosome ten
si: small interfering
SCR: scrambled
VEH: vehicle
REG: regorafenib
ARA: aramchol
MET: metformin
RMA: regorafenib + metformin + aramchol
BID: BH3-interacting domain death agonist
