# Supplementary material for: The SCD1 inhibitor aramchol interacts with regorafenib and metformin to kill tumor cells
Source: Oncotarget. 2026 Mar 27;17:78–89. doi: 10.18632/oncotarget.28861 (PMC13064944; doi:10.18632/oncotarget.28861)
Supplement: Supplementary file 1 [file oncotarget-26-049669-s001.pdf]

The SCD1 inhibitor aramchol interacts with regorafenib and metformin to kill tumor cells

SUPPLEMENTARY MATERIALS

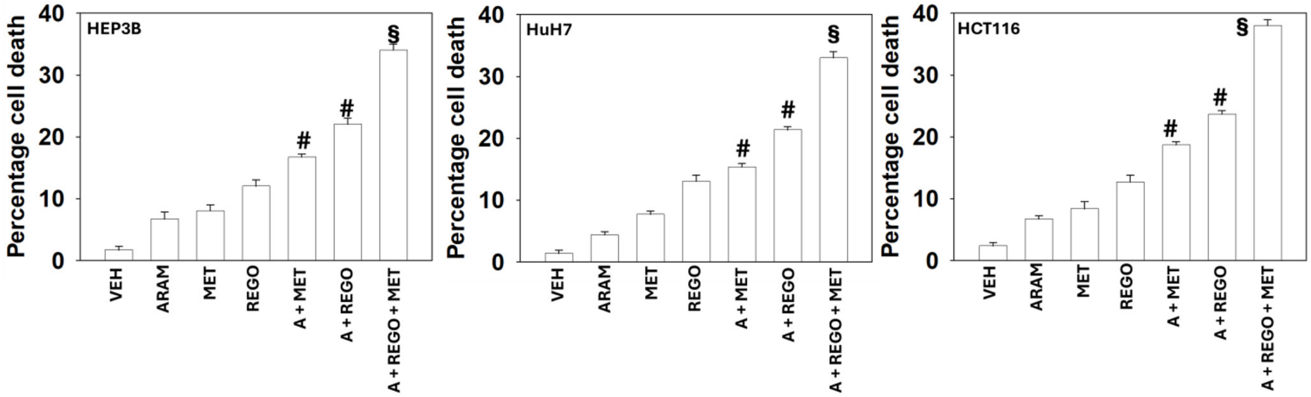

Supplementary Figure 1: Aramchol interacts with regorafenib and metformin to kill GI tumor cells. GI tumor cells were treated with vehicle control, aramchol, regorafenib, metformin, or the drugs combined as indicated for 24 h. Floating and attached cells from three independent studies were collected and the percentage viability determined using trypan blue exclusion assays (±SD). $^{\#}p < 0.05$ greater than cells vehicle control; $^{\#\#}p < 0.05$ greater than cells treated with aramchol; $^{\S}p < 0.05$ greater than cells treated with [aramchol and regorafenib].

Supplementary Table 1: Aramchol, regorafenib and metformin interact to alter the phosphorylation and expression of proteins

HEP3B

4h	VEH	REG	ARM	MET	R+A	R+M	RMA
ATM	100	101	101	101	101	102	101
P-ATM S1981	100	118#	103	111#	119#	121#	124#
AMPKα	100	100	100	101	101	101	101
P-AMPKα T172	100	121#	104	112#	120#	125#	124#
mTOR	100	100	100	100	99	100	99
P-mTORC1 S2448	100	82*	102	90	78*	76*	74**
P-mTORC2 S2481	100	83*	102	90	80*	76*	71**
ERK2	100	100	99	99	100	101	100
ULK1	100	100	99	100	100	99	100
P-ULK1 S757	100	86*	98	91	87*	80*	74**
P-ULK1 S317	100	120#	102#	113#	120#	124#	129##
eIF2α	100	101	101	100	100	99	102
P-eIF2α S51	100	120#	105#	113#	121#	124#	126#
PERK	100	100	100	100	101	99	99
P-PERK T980	100	120#	101#	113#	121#	127#	127#
ERK2	100	100	99	100	100	100	100

	VEH	REG	ARM	MET	R+A	R+M	RMA
AKT	100	100	100	101	99	99	100
P-AKT T308	100	87*	99	93	87*	83*	81**
STAT3	100	99	100	101	100	100	100
P-STAT3 Y705	100	97	99	97	96	96	94
STAT5	100	100	99	100	100	99	100
P-STAT5 Y694	100	95	100	98	94	93	93
ERK2	100	99	99	99	100	100	100
Beclin1	100	115#	100	106	114#	119#	121#
ATG5	100	116#	100	107	115#	120#	121#
ATG13	100	102	101	102	102	101	100
P-ATG13 S318	100	113#	101	104	113#	115#	115#
GRP78	100	117#	101	108	116#	121#	123#
CHOP	100	102	99	104	101	105	105
PP1	100	100	100	102	101	105	104
ERK2	100	99	100	101	101	101	100

HEP3B cells in 96-well plates were treated with vehicle control, aramchol, regorafenib, metformin or the drugs combined for 4 h. Cells were fixed in place, permeabilized and subjected to in-cell immunostaining for the indicated proteins/phosphoproteins. Cells were imaged using an Odyssey infrared imager. ERK2 staining was used as an invariant loading control. The percentage alteration in expression/phosphorylation caused by the drugs was determined from three independent replicates (±SD). $^{\#}p < 0.05$ greater than cells treated with one drug; $^{\#\#}p < 0.05$ greater than cells treated with two drugs; $^{*}p < 0.05$ less than vehicle control; $^{**}p < 0.05$ less than cells treated with two drugs.

Supplementary Table 2: Aramchol, metformin and regorafenib interact to alter the phosphorylation and expression of proteins

HEP3B

4h	VEH	REG	ARM	MET	R+A	R+M	RMA
NFκB	100	99	99	100	100	100	99
P-NFκB S536	100	100	99	99	100	100	100
c-SRC	100	100	100	100	100	100	101
P-SRC Y416	100	99	99	99	99	99	99
P-SRC Y527	100	99	100	99	99	99	100
c-MET	100	100	100	100	100	101	101
P-MET	100	100	100	100	100	100	100
ERK2	100	99	98	99	100	99	99
CD95	100	100	100	100	100	100	99
FAS-L	100	97	100	99	95	96	97
JAK2	100	100	99	99	101	100	98
P-JAK2	100	103	99	98	106	99	99
c-KIT	100	101	100	100	101	101	101
P-KIT	100	97	101	98	95	95	98
ERK2	100	100	100	101	103	100	101

	VEH	REG	ARM	MET	R+A	R+M	RMA
p70 S6K	100	99	100	100	99	100	100
P-p70 T389	100	94	100	97	95	93	89*
JNK1/2	100	99	100	99	99	99	99
P-JNK1/2	100	100	100	94	98	95	96
p38	100	100	99	101	101	100	101
P-p38	100	98	101	94	98	94	95
P-ERK1/2	100	91	102	95	95	91	96
ERK2	100	100	100	100	101	100	100
ERBB1	100	100	100	101	101	100	100
P-ERBB1	100	95	100	99	96	95	95
ERBB2	100	99	100	100	99	99	99
P-ERBB2	100	98	101	96	98	97	95
ERBB3	100	100	100	99	100	100	100
P-ERBB3	100	92	89*	92	90*	92	90*
ERK2	100	100	99	100	100	100	100

HEP3B cells in 96-well plates were treated with vehicle control, aramchol, regorafenib, metformin or the drugs combined for 4 h. Cells were fixed in place, permeabilized and subjected to in-cell immunostaining for the indicated proteins/phosphoproteins. Cells were imaged using an Odyssey infrared imager. ERK2 staining was used as an invariant loading control. The percentage alteration in expression/phosphorylation caused by the drugs was determined from three independent replicates. (±SD). $^{\#}p < 0.05$ greater than cells treated with one drug; $^{\#\#}p < 0.05$ greater than cells treated with two drugs; $^{*}p < 0.05$ less than vehicle control; $^{**}p < 0.05$ less than cells treated with two drugs.

Supplementary Table 3: Aramchol, metformin and regorafenib interact to alter the phosphorylation and expression of proteins

HEP3B 4h	VEH	REG	ARM	MET	R+A	R+M	RMA
ERBB4	100	99	99	99	100	99	98
P-ERBB4	100	97	101	95	93	95	95
BCL-XL	100	94	101	97	93	90*	88*
MCL1	100	92	101	95	88*	87*	85*
BAX	100	104	100	103	104	106	108
BAK	100	103	99	105	103	107	107
BIM	100	102	103	103	101	106	117#
ERK2	100	99	99	99	100	100	99
LATS1/2	100	100	100	100	100	101	100
P-LATS T1097	100	101	99	101	101	103	104
P-LATS S909	100	102	101	102	100	102	103
YAP	100	99	99	100	99	100	100
P-YAP S109	100	99	99	96	99	96	94
P-YAP S127	100	104	100	99	102	99	103
P-YAP S397	100	102	99	96	101	98	102
ERK2	100	99	99	99	99	100	99

HEP3B cells in 96-well plates were treated with vehicle control, aramchol, regorafenib, metformin or the drugs combined for 4 h. Cells were fixed in place, permeabilized and subjected to in-cell immunostaining for the indicated proteins/phosphoproteins. Cells were imaged using an Odyssey infrared imager. ERK2 staining was used as an invariant loading control. The percentage alteration in expression/phosphorylation caused by the drugs was determined from three independent replicates (±SD). $^{\#}p < 0.05$ greater than cells treated with one drug; $^{\#\#}p < 0.05$ greater than cells treated with two drugs; $^{*}p < 0.05$ less than vehicle control; $^{**}p < 0.05$ less than cells treated with two drugs.

Supplementary Table 4: Control data demonstrating the percentage siRNA knock down of proteins

	ATM siSCR	ATM siProtein	AMPKα siSCR	AMPKα siProtein	LAMP2 siSCR	LAMP2 siProtein	Beclin1 siSCR	Beclin1 siProtein	ATG5 siSCR	ATG5 siProtein	CD95 siSCR	CD95 siProtein	FADD siSCR	FADD siProtein	SCD1 siSCR	SCD1 siProtein
Protein	100	26	100	20	100	26	100	28	100	30	100	25	100	32	100	22
ERK2	100	100	100	100	100	101	100	100	100	100	100	101	100	100	100	100

TPF-16-75 cells were transfected with a scrambled control siRNA (siSCR) or with an siRNA to knock down each specific protein, as indicated. After 24 h, cells were fixed in place an in-cell immunostaining performed to detect the protein levels of each protein, as indicated. Parallel staining for ERK2 was performed as a loading control (*n* = 3 independent assessments, ±SD).
